# A socioecological examination of father alcohol use in Kenya: Motivation, consequences, and barriers to care

**DOI:** 10.1080/17441692.2025.2515481

**Published:** 2025-06-09

**Authors:** Ali Giusto, Emily N. Satinsky, Florence Jaguga, Wilter Rono, Julius Barasa, Chardée A. Galán, Milton L. Wainberg

**Affiliations:** a Department of Psychology, Florida International University, Miami, FL, USA; b Department of Psychology, University of Southern California, Los Angeles, CA, USA; c Department of Alcohol and Drug Abuse Rehabilitation Services, Moi Teaching & Referral Hospital, Eldoret, Kenya; d Department of Mental Health, Moi Teaching & Referral Hospital, Eldoret, Kenya; e Department of Psychology, Pennsylvania State University, University Park, PA, USA; f Department of Psychiatry, Columbia University Medical Center, New York State Psychiatric Institute, New York, NY, USA

**Keywords:** Alcohol, fathers, Kenya, barriers, motivation

## Abstract

Fathers’ alcohol use impacts family well-being, including increased risk for violence, poor child outcomes, and low engagement in care. Yet few studies examine the drivers of alcohol use among fathers or the role of gendered expectations and sociocultural norms on use, especially in low-resource settings like Kenya. Understanding why fathers drink, the consequences of use, and barriers to care is key to designing scalable, responsive interventions. In Eldoret, Kenya, community members, leaders, providers, and fathers experiencing problematic alcohol use participated in interviews and focus groups. Participants discussed reasons for drinking, its impacts, and barriers to care. Using the framework method, transcripts were coded and summarised using the socioecological model. Reasons and consequences of alcohol use emerged across individual, interpersonal, and sociocultural levels. Individually, fathers used alcohol to escape distress with consequences on physical and mental health. At the family level, alcohol was used to avoid conflict, contributing to risk for violence and poor child outcomes. Socioculturally, drinking was shaped by gender norms, with consequences like stigma and loss of social status, which reinforced shame and isolation. Barriers to care included lack of awareness, poor service access, and stigma. Intervention and implementation strategies must address avoidant coping, masculinity norms, and local resource constraints.

## Introduction

Globally, alcohol use disproportionally impacts men. Individual and family consequences of alcohol use account for 9.6% of disability adjusted life years (Rehm et al., 2009, 2010). In Kenya, men are disproportionately affected, with 15.7% of men and 4.6% of women meeting criteria for an alcohol use disorder (NACADA, 2022). Kenya also has the third highest total disability-adjusted life years from alcohol use disorders (AUD) in Africa (Degenhardt et al., 2018).

Problematic alcohol use – including both diagnosable AUDs and harmful drinking patterns – frequently co-occur with mental health conditions like depression and anxiety. Problematic alcohol use is sometimes considered a diffuse marker of mental health concerns (Jane-Llopis & Matytsina, 2006; Kendler et al., 2003). This is especially relevant for men, who tend to underreport internalising symptoms like depression (Sigmon et al., 2005). Alcohol use is also linked to heightened risk of mortality, cancer, poor mental health, and violence (Carvalho et al., 2019; Roerecke & Rehm, 2013).

The consequences of men’s alcohol use extends to families (Flouri, 2010; Sigmon et al., 2005). Alcohol use among men, particularly fathers, can disrupt family systems and negatively impact couple relationships, parent–child relationships, and the mental health of other family members (Goeke-Morey & Mark Cummings, 2007; Rossow et al., 2015). While some studies have explored contributing factors and consequences of alcohol use among fathers (Dimova et al., 2022), most research comes from high-income countries the United States. In contrast, these issues remain understudied in low-and-middle-income countries (LMICs) such as Kenya (Jaguga & Kwobah, 2020).

More research is needed in LMICs to understand the specific dynamics of father alcohol use and its potential effects on families (Giusto et al., 2021; Jaguga & Kwobah, 2020; NACADA, 2022). Furthermore, fathers are central to family and family member well-being, but they are often missing from the family-focused literature (Flouri, 2010; Giusto & Puffer, 2018). In Kenya and globally, mothers are more commonly engaged in mental health and parenting research, leaving a gap in understanding fathers’ experiences and needs (Panter-Brick et al., 2014).

To effectively engage and intervene with fathers, we must understand the motivations for fathers’ alcohol use, its mental health and relational consequences, and the barriers to seeking help. Qualitative methods provide an opportunity to explore how community members perceive both the motivations for and consequences of fathers’ alcohol use, as well as the barriers that limit help-seeking. For example, some evidence from Kenya suggests that while fathers may accept informal support from family or community members, they rarely access formal services. Factors such as low awareness, stigma, and limited availability of care hinder help-seeking. Identifying and specifying these barriers from a community perspective can inform strategies that improve access and engagement in treatment. Further, community perspectives can inform the framing and contextualisation of treatment approaches that often focus on psychoeducation and assessment of drinking motivations to replace those behaviours (Yeh et al., 2017). Qualitative data can offer rich, contextual insights into the dynamics around fathers’ alcohol use and guide the development of responsive, community-grounded interventions.

This study aimed to: (1) explore the motivations behind alcohol use among fathers in Eldoret, Kenya; (2) identify the consequences of their alcohol use; and (3) describe barriers to seeking help. A secondary aim was to examine how these findings vary across socioecological levels (individual, interpersonal, sociocultural). A tertiary aim was to explore insights to inform clinical and implementation strategies to support fathers and families.

## Methods

### Study design and approach

We conducted key informant interviews (KIIs) and focus group discussions (FGDs) with community members, leaders, mental health providers, and individuals experiencing problematic alcohol use. This qualitative study was part of a larger effort to identify implementation determinants for delivering a mental health and alcohol use intervention for fathers in the area (Giusto et al., 2023).

#### Interview Guides.

Interview guides for the full study were informed by the Consolidated Framework for Implementation Research (CFIR) and the Integrated Sustainability Framework (ISF) (Damschroder et al., 2009; Shelton et al., 2018). These frameworks helped structure questions by domains such as outer-level (i.e. culture, stigma, country), inner-level (i.e. settings like hospital or community), and individual-level (i.e. characteristics, attitudes, and beliefs of providers and patients) influences. The interview guide included questions on the following: perceived causes and motivations of and for father alcohol use (e.g. ‘*Tell me about any feelings, situations, or cultural beliefs that may lead to fathers drinking*’); consequences of alcohol use (e.g. ‘*What are the biggest problems you see in the family caused by fathers’ alcohol use?*’); and barriers to help-seeking (e.g. ‘*What gets in the way of fathers making changes or getting help?*’). Questions included probes aligned with each CFIR and ISF domain. The study is reported following the Standards for Reporting Qualitative Research (Appendix Table 1).

### Ethical approval and setting

KIIs and FGDs were conducted in Eldoret, Kenya, in partnership with the Kenyan Ministry of Health, Moi Teaching and Referral Hospital (MTRH), and AMPATH, a collaborative service and research entity comprising MTRH and a consortium of North American educational institutions (https://www.ampathkenya.org/). Ethical approval was obtained from the Institutional Review Board at New York State Psychiatric Institute (Protocol 8084) and the Institutional Ethics Review Committee at Moi Teaching and Referral Hospital (Approval Number 0001138).

### Participants

Participants included individuals with a range of experiences with alcohol use and mental health treatment in the area. A total of 18 individual KIIs and eight FGDs (N = 31 participants) were conducted. KII participants included four community leaders, three policy makers, five hospital leaders, three individuals who received alcohol use treatment, and three peer-father counsellors (i.e. lay counsellors trained to provide alcohol use treatment). FGDs were conducted with fathers experiencing alcohol problems (6 participants, 1 FGD), mental health providers (21 participants, 3 separate FGDs), and community leaders (4 participants, 1 FGD).

The study sought a diverse selection of participants, including individuals from various sectors, both rural and urban, and lay and professional backgrounds. Individuals currently experiencing problematic alcohol use were eligible if they were fathers responsible for children under 18, exhibited problematic alcohol use (indicated by Alcohol Use Disorder Identification Test, score of 8–19 (Babor et al., 2001) administered during screening), and had engaged in alcohol use within the past two months.

### Recruitment

Potential participants were approached and consented by a trained Kenyan research assistant (RA) or the project coordinator. Community leaders, policy makers, hospital leaders, and providers were identified based on study team knowledge and consultation with local leaders; they were contacted by phone, email, or in person to assess initial interest. If the individual expressed interest, the RA or project coordinator obtained consent. A full description of procedures can be found in (Giusto et al., 2023). All participants were included if they were interested and consented to participate.

Previous patients and peer-father counsellors were identified through records of a previous project led by the lead author (Giusto et al., 2022). All three providers from the previous study were contacted. For former participants, identification numbers were randomly assigned a value using the Microsoft Excel random number generator. Those assigned numbers 1–4 were invited first; if they declined or were unreachable, those assigned numbers 5–9 were subsequently contacted. The RA called or met participants to provide a brief overview of the study and assess initial interest. If a participant expressed interest, the RA obtained written consent in person.

Current patients were recruited when presenting to MTRH or affiliated support groups. They were approached by an RA who explained the study. If individuals expressed interest, the RA completed the screening process, assessed alcohol use, and consented eligible fathers. Fathers who did not meet eligibility criteria were assured that they would continue receiving care with MTRH.

### Procedures

This study took place between 2021 and 2022. Data collection was conducted by Kenyan RAs in collaboration with the on-site investigator. The on-site investigator had a relationship with some of the policy makers and mental health providers. In these cases, the interview was conducted by the team member who knew the participant least. KIIs and FGDs were conducted in English or Kiswahili based on participants’ preferences. KIIs typically lasted around 45 min, while FGDs were longer and typically took between two and three hours. All sessions were recorded, deidentified, and transcribed into English. Specific Kiswahili idioms were maintained in the transcripts as appropriate. (The full interview guides are available in the larger study paper, (Giusto et al., 2023)).

### Analysis

Qualitative analysis was guided by the framework method (Gale et al., 2013). The framework method belongs to a broader group of content analysis approaches and can accommodate deductive, inductive, or combined qualitative analytic approaches. Typically following a seven-stage process, data are grouped into codes that are then used for data organisation and interpretation. In this study, we incorporated both predefined and data-driven codes based on the questions asked. Although the overarching themes – reasons for fathers’ alcohol use, consequences of alcohol use, and barriers to help-seeking – were deductively aligned with the primary study aim, the sub-themes emerged inductively through the coding and data synthesis process. While the CFIR and ISF frameworks guided the development of the interview guides for the larger qualitative study, Bronfenbrenner’s ecological model (Bronfenbrenner, 1994) emerged as a more fitting framework to organise themes and interpret data for these research questions given their focus on father behaviours and consequences rather than implementation of an intervention. The ecological model describes how individual behaviours and experiences are shaped by immediate contexts (e.g. family, peer relationships; microsystem) and socio-cultural structures (e.g. cultural ideologies, social norms; macrosystem). The domains of this model parallel those of the implementation frameworks, yet the outcome of interest differs.

The analysis team included a Kenyan psychiatrist, a Kenyan research manager, and a US-based psychologist. This allowed us to interpret and balance data from multiple, diverse perspectives. The team read the transcripts and took notes in memos. The team then discussed notes to generate broad codes. Next, the lead author selected a subset of interviews by participant type from within each interview type (e.g. KII or FGS). Individual team members coded these selected interviews. Use of codes and transcripts were discussed to develop a coding framework and codebook. Transcripts were coded independently until 80% agreement was met across four coders. Since percent agreement is directly interpretable (McHugh, 2012), we chose this method to determine when to move to independent coding. Next, transcripts were divided and coded independently using NVivo 12.0; questions that arose were discussed to reach consensus. Coded data were then reviewed and summarised. Lastly, we interpreted data. As the study aims focused on the reasons and consequences of alcohol use as well as barriers that might influence these factors, data were organised by these codes. Codes were synthesised with reference to transcripts as needed. Notably, some participants discussed patterns of alcohol use among men as a whole, rather than among fathers specifically. Because these patterns also apply to fathers and inform clinical and implementation strategies aimed at engaging fathers in care, all participant perspectives were included during the coding process.

## Results

**Figure 1.**
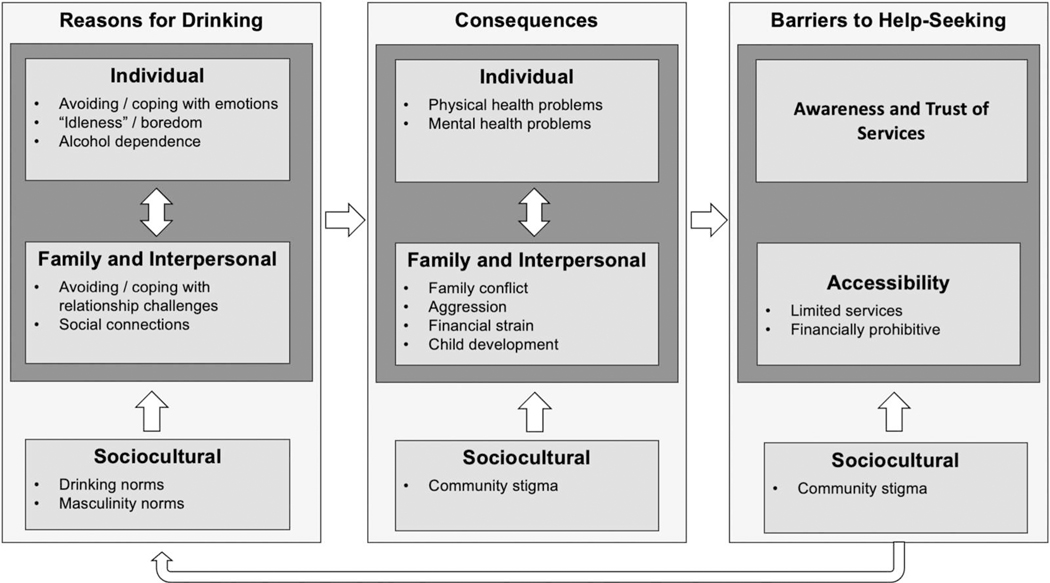
Interrelated cycles of reasons for drinking, consequences, and barriers to help seeking among fathers in Kenya. Note: Figure depicts relationship between the reasons for drinking, consequences of use, and barriers to help seeking.

Across participants, multiple interrelated factors were described as driving father’s alcohol use and the consequences of use. Although alcohol use treatment services exist in the region, participants described barriers that stopped fathers from seeking and accessing care. These barriers appeared to maintain and reinforce patterns and consequences of use. Figure 1 provides an overview of these results, guided by Bronfenbrenner’s ecological model (Bronfenbrenner, 1994). This figure illustrates the cyclical and reinforcing nature of the factors at different ecological levels. Below, we first describe noted reasons for drinking, and then we describe consequences of fathers’ alcohol use. These themes are organised by ecological levels (individual, family/interpersonal, sociocultural). We then present barriers to treatment that specifically impacted fathers’ reasons for drinking and maintained consequences of use.

### Reasons for drinking

Reasons for fathers’ drinking were noted at multiple levels. The function of drinking – i.e. to avoid emotion or connect – was often similar across these levels. At the individual level, stressors included mental health problems, ‘idleness’, and dependence. Interpersonal factors included family and peer conflict as well as social connection. Sociocultural drivers included norms around drinking and cultural expectations regarding masculinity. We organise results by these levels.

#### Individual.

Participants often perceived mental health problems or difficult emotions as key drivers of alcohol use. Specific concerns included depression, anxiety, stress, trauma, anger, grief, and poor coping skills and the use of alcohol as a means to escape these problems (i.e. avoidant coping). Many participants noted that alcohol use to escape mental distress can be driven by financial stress occurring in a context of poverty and expectations to financially provide. One community leader observed among fathers in the community, ‘*you find that someone is worried. They have a lot of bills to take care of,* [and] *the relationship problems … they want to forget about all these problems, and so what do they do? The next solution is alcohol*’ (FGD 101 – P1). Similarly, two fathers currently in treatment reflected on their own experiences of using alcohol to cope with anger. One described, ‘*something small can make me angry. You run away telling yourself that you want to go and take away the anger*’ (KII 1603). However, this same patient noted that alcohol does not fix the anger ‘ – *you get angry again*.’

Related, drinking to cope with idleness or boredom emerged as a key theme. Participants described how boredom and feeling a lack of purpose, often in the context of unemployment, led fathers to drink. Some participants noted how COVID-19 amplified this problem. Many fathers lost work during the pandemic and turned to alcohol to cope with ‘idleness’. Reflecting on their perception of fathers’ reasons for drinking, one participant described, ‘*They do not have anything to do in terms of work. Most of them are jobless and so they wake up in the morning and … they tend to take alcohol because they do not have anything to do*’ (KII 1302 – Clinical Officer).

Lastly, physical dependence on alcohol, or reliance on alcohol to function, was noted as both a reason and consequence of alcohol use. Patients and providers described how fathers might start drinking as soon as they wake up to get energy to start the day.

#### Family and Interpersonal.

Challenges in fathers’ family and peer relationships were also cited as reasons for drinking. Similar to dealing with negative emotions, participants described how fathers might use alcohol to avoid conflict and forget quarrels with partners or difficult interactions with their children. For instance, one community leader observed: ‘*Marital conflict is a key thing. And once you have had conflict, you do not wish to go back to the house and face the same woman you fought, and so what do you do? You go there to the* [alcohol woman] *and forget yourself for a moment*’ (FGD 101 – P1). Conflicts outside the immediate family were also noted as potential reasons for drinking. A counselling psychologist, for example, commented, ‘*The other challenge is … the relationship at the workplace … and in the community generally*’ (KII 1309).

While many participants viewed alcohol as a way to escape, some participants shared that fathers might use alcohol to handle problems directly. These participants reflected that some fathers feel more comfortable confronting their wives or talking through disagreements when they are drunk. A current patient described their experience, ‘[I] *cannot confront the problem when I am not drunk*’; the same patient added that when he is drunk, ‘*Whatever was bothering me will all come out … When you drink alcohol, you say it all*’ (FGD 701 – P3). Other participants discussed a similar sentiment in reference to peers – alcohol gives men the courage to confront community members who wronged them or talk openly with peers about their marital stress.

Lastly, alcohol use was described by some as a means of social connection, such that a reason for drinking may be celebrating or being with friends. A psychiatrist described their perception of these phenomena saying, ‘*It is like a support group kind of … so they form these cocoons, and so these cocoons propel the drinking. They help each other drink. If you do not have money, I will buy for you today, if you have tomorrow, you will buy for me … so they form a cocoons of similar behavior. They live with those cocoons until when they have advanced issues*’ (FGD 401 – P3).

#### Sociocultural.

Outer-level factors related to gender norms and drinking norms emerged as perceived drivers of father alcohol use. These intersected with and compounded individual and interpersonal level drivers by reinforcing and contributing to motivations for using alcohol as a means to escape or cope with distressing emotional experiences.

##### Drinking norms.

Participants described two social factors that may promote fathers’ alcohol use: peer pressure and cultural events tied to alcohol. First, one participant observed, ‘*we take it for granted that the peer pressure affects the youth alone, but I have seen it affect even men*’ (FGD 101 – P2 youth student leader). Men engage in alcohol use when they see it modeled in others, with one patient reflecting, ‘*That issue of being* [the] *odd one out can become difficult*’ (FGD 702 – P3). Some peer pressure may, in part, be driven by cultural norms around men’s drinking. An addiction specialist described these norms – ‘*culturally, you are not supposed to say that I cannot drink*’ (KII 1203). A psychiatrist added their perception, ‘*in the norms of many societies, they feel that if a man does not drink, then he is the odd one out*’ (KII 1313).

Second, during certain events, alcohol is widely available, and drinking is encouraged among men. In some areas, such as the urban informal settlements, boys and young men sell alcohol to earn a living. They may drink ‘*because it is just there in plenty*’ (FGD 401 – P1 social worker). This participant observed that the presence of alcohol in these settings means that drinking often passes down through generations. Another participant mentioned that Friday is a drinking day in the community, while several others added that alcohol is embedded into ‘circumcision time’ and other rituals. Alcohol use norms during these ceremonies can amplify social pressures to drink.

##### Masculinity Norms.

Norms around masculinity emerged as a specific reason that fathers might use alcohol to cope with distressing emotions. Participants repeatedly discussed expectations that fathers appear strong, act ‘macho’, or have ‘alpha energy’. As a clinical psychologist described, ‘*There is the cultural aspect where men have himself together. Man is not supposed to say that I am overwhelmed*’ (KII 1203). Expectations that men never show weakness might compound feelings of shame and loneliness, and thereby prompt engagement in alcohol use. As gender norms are deeply ingrained in society, fathers often lack role models for how to deal with stress and difficult emotions in healthy ways. Men see examples of women discussing mental health problems but do not see such modeling among other men. When individuals approach their own fathers, cultural beliefs might be reinforced:

*I think it is harder for our fathers to approach their fathers. This is because when you look at those older men, they are those kind of people who are closed off … They will tell you to ‘struggle like a man’, and so the moment you lack a role model, you will go to your fellow man whom you know … the first thing he will do is go to the bar, and so you will follow each other to the bar*. (FGD 101 – P2 student leader)

Given this cultural context, men ‘*just lock it in*’ and might turn to alcohol use and other harmful behaviours as an outlet for their emotions.

Many participants described how these problems start with ‘*negligence of the boy child*’. They noted that while significant funding has gone into supporting and empowering girls, boys get left behind. A psychiatric nurse elaborated on their perception:

*We are putting more emphasis on girls; we are protecting girls more than we are protecting boys. During the growing up of the boy, we are telling them that the boy does not cry and therefore we are telling them untrue and then we are not preparing them for the challenges that are there right now … And then because of this empowerment of the girl child, the boy has lost his identity. Now, when he becomes a man or when he becomes a father or a husband, the nature of the man, is already undermined … now they enter into … alcohol. They go in groups of men and from there they feel better, they feel accepted, when they are in a group and then they are drinking. But they do not know that that kind of drinking has its own steps from curiosity up to the time the person is hooked he cannot go back. The challenge started from when he was a boy*. (FGD 401 – P4)

This quote highlights how masculine norms, social connection (interpersonal), and ‘acceptable’ coping skills, in this case alcohol use for distress (individual), can also intersect to influence and maintain men’s alcohol use. Further, participants noted that efforts to empower girls have shifted norms. A psychiatrist and social worker both commented on how women no longer depend on men financially. These changes can instill a disconnect between perceptions of societal gender roles (i.e. men should provide) and actual behaviours (i.e. women earn more). As a psychiatrist suggested, this cultural conflict can further contribute to alcohol use among fathers.

### Consequences of drinking

As with the drivers of fathers’ alcohol use, the reported consequences of drinking also spanned multiple ecological levels. Participants discussed negative impacts at the individual, interpersonal, and sociocultural levels.

#### Individual.

At the individual level, consequences included physical and mental health problems. Physical health consequences included direct effects of alcohol use on health, such as liver cirrhosis, cancer, and death. Participants also mentioned indirect effects. For example, when fathers drink, they are less likely to maintain their hygiene, thus allowing opportunistic diseases. Furthermore, many noted drinking can increase likelihood of risk behaviours that increase susceptibility to COVID-19, HIV, and other sexually transmitted infections.

Discussing mental health, participants described how alcohol use can contribute to shame, depression, and anxiety among fathers. A consultant psychiatrist commented, ‘*and then anxiety related to alcohol use – predisposing and also an outcome from alcohol use*’ (KII 1206). Fathers’ lives become unmanageable, and they feel powerless to cope with daily life. Some providers observed that this hopelessness can also motivate suicidal ideation and behaviours.

#### Interpersonal.

Participants universally noted that fathers’ excessive alcohol consumption can lead to family conflict. Several participants perceived that problem alcohol use among fathers contributes to potential for physical and emotional aggression in the family, escalating at times into violence. A church leader described their observations: ‘*before they are drunk, they are just good people … but after drinking, they become violent. They become abusive*’ (KII 1103). Risk of emotional and psychological abuse was also noted. Living in this environment, ‘*children live in fear, women live in fear*’ (FGD 102 – P1 church leader). Some women threaten to leave, leading to ‘*breakage of the marriage*’ (KII 1104 – advisory committee member).

Financially, fathers’ alcohol use constrains the availability of resources for essential needs within the family. Participants shared that when fathers engage in problematic alcohol use, families often struggle to cover expenses for rent, school fees, medical services, seeds and fertilisers, soap, food, and sugar. As one community provider explained, some fathers even resort to selling items from their household in exchange for money to fund their drinking. A father seeking treatment for his alcohol use reflected that ‘[you are] *entertaining yourself as your children struggle*’ (KII 1603). A village elder perceived that when families are restricted in these ways, they seek money from other sources: ‘*the mother will result into looking for money from another man … The children can go stealing so that they can get something to eat’* (KII 1101).

Alcohol use was also reported to indirectly impact financial stress through professional problems. More specifically, alcohol use may result in fathers missing work, which not only threatens a family’s capacity to fulfil their essential needs on days when fathers are absent but also heightens the risk of long-term unemployment. Losing a job then amplifies conflict and financial stress in the family. A provider observed the following pattern:

*Most employers do not understand alcoholism as a disease, and they sack you. When they sack you, you go back to the house and your wife doesn’t want to understand.* [She] *wants to blame you for being irresponsible and abusing … your wife abandons you and even sends you away from your family because even now, you are good for nothing husband and now you get into economic hardship*. (KII 1202)

Interviews highlighted the ways in which youth are affected by their fathers’ alcohol use and the resulting family conflict that it provokes. Participants shared that children in these environments lack confidence and feel depressed. Many children do not go to school consistently due to lack of school fees and familial stress. When they *do* attend school, they may perform poorly. As a church leader considered, ‘*even if they go to school, they cannot afford to go to school five days in a week … you find that in such areas, once a man has lost focus in the family, everything scrambles in the family and in the neighborhood*’ (FGD 102 – P1). In addition to poor school attendance and performance, some children show behavioural problems. A father in treatment for alcohol use reflected on his experience: ‘*First it hurts the children, because these children will not have discipline because when the father is not around, sometimes a mother has to deal with grown up boys. They will not respect the mother the way they respect the father. So, the children will break the rules … they become poorly disciplined.*’ (FGD 702 – P2).

#### Sociocultural.

When fathers’ alcohol use advances to more severe levels, there can be consequences in the community. Fathers may be stigmatised, lose social status, and get ‘*left behind*’. Referencing the loss of status, a mental health provider observed, ‘*and that loss is not easy to navigate … they rather resign to fate of being called a drunkard because they can no longer stand up to pick up their position*’ (FGD 401 – P5). A psychiatrist elaborated on their observations, describing the process by which men experiencing problems with alcohol use are pushed to the outskirts of society: ‘*they are shunned right from the working areas to the community activities, leadership roles in the society … they are not valued anymore. They feel that they are appendages in the community building*’ (KII 1313). In this context, fathers may feel shame and further self-isolate to avoid societal humiliation. A father receiving treatment for alcohol use personally reflected, ‘*you look like a bad man, and when a man looks bad … do you know that they will throw you away? … Things become so bad*’ (FGD 701 – P2). Given expectations around masculinity, there appears to be little cultural empathy for men with mental health and alcohol use problems.

### Barriers to care and maintenance of the cycle

Participants listed several barriers that stand in the way of men accessing alcohol use treatment or other forms of care. These barriers maintain and reinforce drinking habits and exacerbate consequences in a cyclical manner.

#### Lack of awareness and trust.

Among fathers, lacking awareness of the problem and available services were barriers to help-seeking. Two psychiatrists perceived that fathers are often unable to recognise when alcohol use interferes with their life. A third psychiatrist reiterated this, suggesting that the lack of awareness may be due to cultural beliefs: ‘*You know sometimes we have cultural explanation of challenges. If you have a challenge, instead of seeing it as a health care issue, you see it as you*’ (KII 1308). If fathers think that their behaviours are normative in context, they may be unlikely to seek care.

A hospital manager added that fathers might also lack knowledge of available services to support them in addressing their problematic alcohol use. While many women-specific health services exist, men often do not know of similar services tailored to men and may assume services are non-existent. When fathers *do* know about services, a lack of trust may emerge as a further hindrance to help-seeking. Some men might feel like the services cannot help them while others might feel wary sharing sensitive information. For example, a psychologist reflected on their experience providing care: ‘[fathers] *do not really want to express some of the issues, especially with marriage. They do not want to talk about it*’ (FGD 403 – P3).

#### Accessibility barriers.

Even when fathers are aware of services, providers noted that access is limited. One provider noted that the establishment of alcohol use programmes is not prioritised. Distance to existing treatment programmes further impeded access. Lastly, the cost of treatment arose as the most prominent accessibility concern. Some participants suggested that, even if treatment costs are reasonable, fathers might prefer to spend that money on their family. A hospital manager elaborated on this perception: ‘*it will be down the ladder … because the child has to go to school, let me first sort out the school fees. We are lacking food here, and then, by the time you reach to yourself, I cannot pay for that service, so I will survive like a man*’ (KII 1301). A psychologist added, ‘*they will feel that they are leaving a gap at home … who is paying the rent for those three months while I am in rehabilitation in the inpatient care?*’ (FGD 402 – P1). A couple participants also mentioned potential issues with insurance.

#### Social stigma.

Stigma was identified as a critical barrier to help-seeking. A psychiatrist suggested that drinking is not seen as a health problem but rather as a moral issue. Others described stigma surrounding the idea that people who choose to drink bring problems upon themselves. At the same time, policies that criminalise drug use and suicide make help-seeking particularly difficult for individuals with comorbid problems. In this context, fathers might avoid care.

Social stigma is exacerbated by gender norms, limiting help-seeking among men. Referencing cultural expectations around masculinity, several participants referenced observations that men are perceived as weak if they access mental or behavioural health treatment – ‘*you are a less of a man according to them if you go*’ (KII 1309 – counselling psychologist). A rehabilitation provider summarised,

*We are in a patriarchal environment whereby a man is supposed to be the provider … So now when a man has mental issues or is a man struggling with alcoholism, it means that the leadership role has demeaned … The stigmatizing words you hear from colleagues, friends, and family about this person … they take away the ego of a man … How can you actually confess that you are an alcoholic? I think stigma has been the main challenge for men to seek for services*. (KII 1202)

A mental health provider echoed the idea that men are supposed to be the ones with the solutions to problems rather than the ones with the problems themselves. These ideas often become internalized as shame self-stigma, further contributing to a lack of help-seeking. A youth student leader further described this perception: ‘*most of them believe that help is for the weak and no one wants to be seen as weak … you know men have an ego, and so the moment you go to someone asking for help … they will say I have failed*’ (FGD 101 – P2).

## Discussion

This study used qualitative methods to examine the motivations behind alcohol use among fathers in Eldoret, Kenya, the consequences of use, and barriers to help-seeking. Findings highlighted how these factors interact across socioecological levels – individual, interpersonal, and sociocultural – and reinforce each other in a cyclical manner. Key motivations included avoidant coping for emotional and financial stress and alcohol to facilitate social connection. Consequences included negative impacts on fathers’ health, family conflict, and child distress. Barriers to care – particularly limited awareness, stigma, and access challenges – further sustained harmful use patterns. Results can inform both clinical and implementation strategies to better engage fathers and address alcohol-related harm.

Fathers’ alcohol use was motivated by both negative (e.g. escape distress) and positive reinforcement (e.g. enhance social connection), reflecting established evidence on avoidant and social coping patterns. Intervention efforts can address specific drinking motivations and teach alternative coping strategies, as commonly emphasised in cognitive behavioural therapy. Family conflict as a trigger and consequence of alcohol use further reinforces the need for integrating family-based assessment and treatment into approaches. Evidence from high-income settings suggests that treating caregiver mental health can improve family outcomes (Cuijpers et al., 2015), and couples-based interventions have shown promise for reducing alcohol use and improving relationships (Lam et al., 2009). Adapting such approaches to local contexts may prove fruitful for improving father engagement in mental health care and the effectiveness of treatment.

Gender norms around masculinity strongly shaped drinking behaviour, consequences, and barriers to care. Rigid expectations to be a provider and not express vulnerability contributed to distress, alcohol use, and avoidance of help-seeking. These patterns mirror prior findings in Kenya (Giusto et al., 2022; Patel et al., 2020) and globally (Gordon et al., 2013). Highlighting the intersection of masculinity and social norms on behaviour, for example, a study in Uganda found that men who perceived male peers to drink were more likely to drink themselves (Perkins et al., 2022). At the implementation level, engaging men in care may require framing treatment in ways that align with masculinity norms and working with peers to model healthy behaviours. For example, recruiting peer fathers to deliver services may reduce stigma and reinforce masculine norms of strength through care-seeking (Berger et al., 2013; Giusto et al., 2021). Within treatment, it is important to address how internalised gender norms might drive distress and interfere with coping (Giusto et al., 2022; Shafer et al., 2019). Economic stress and unemployment further compounded these issues, suggesting that combining alcohol interventions contextualised for gender norms with economic strengthening or vocational training could further enhance the relevance and impact of treatment approaches.

Interpersonal consequences of drinking were pronounced, particularly for families. Fathers’ alcohol use affected family dynamics through increased conflict, reduced financial stability, and diminished child well-being. These findings are consistent with literature linking paternal alcohol use to youth mental and behavioural health challenges (Andreas & O’Farrell, 2007; Landberg et al., 2019; Thor et al., 2022). Participants also noted that alcohol use contributed to poor financial decision-making, with resources diverted from essential needs. These dynamics reflect prior studies in Kenya (Patel et al., 2020) and underscore the value of including financial tracking or empowerment components in treatment. For instance, fathers in a behavioural activation intervention that incorporated financial tracking (i.e. money saved and spent on alcohol) alongside tracking mood and alcohol use reported that seeing money saved from reduced drinking motivated them to continue treatment (Giusto et al., 2020).

Stigma was also a key cross-cutting barrier, shaped by social and gender norms. Stigma surrounding mental health and alcohol use, particularly perceptions of weakness tied to seeking help, discouraged fathers from engaging in care. This aligns with prior findings across global contexts (Corrigan, 2004; Keyes et al., 2010). Another qualitative study of new fathers similarly found that stigma and pressure to conform to masculine norms stood as barriers to fathers seeking help (Pedersen et al., 2021). Recommendations for addressing stigma include increasing awareness using strategies that involve education and sensitisation, community advocacy and mobilisation, and/or universal advertising of meetings for all people regardless of whether they are impacted by alcohol use (Rao et al., 2019; Thornicroft et al., 2016). A study in Uganda demonstrated that although women are more likely than men to attend community sensitisation meetings, they likely pass along meeting information to men through informal networks (Kakuhikire et al., 2021). Other strategies include working with individuals with lived experience (e.g. fathers in recovery) to deliver programmes and lead national campaigns focused on stigma reduction.

This study has several limitations. First, some themes may apply broadly to men, not just fathers. However, we maintained a focus on fathers given the study’s aims and the emphasis on family impacts in participants’ narratives. Nonetheless, study insights may also inform interventions targeting alcohol use among men more generally. Second, fathers with problematic alcohol use in the sample had either previously received or were currently receiving care. This limits our understanding of how fathers who have not received care perceive motivations for alcohol use, the consequences of such use, and barriers to care. Relatedly, the sample did not include partners or children of men experiencing problematic alcohol use, which could provide additional perspectives.

## Conclusion

This study contributes to a growing body of literature examining the intersection of gender, mental health, and substance use in low-resource settings. By centreing community voices – fathers, providers, and leaders – this work highlights how avoidant coping, masculine identity, and structural barriers intersect to shape fathers’ alcohol use and related consequences. Results can inform multifaceted intervention and implementation approaches to engage fathers in services and effectively treat alcohol use and mental health problems Clinically, brief psychosocial strategies (e.g. behavioural activation, motivational interviewing) and family-inclusive formats may support change. Implementation strategies might prioritise stigma reduction, peer-delivered care, and integration into community-based settings.

## Supplementary Material

Appendix

Supplemental data for this article can be accessed online at https://doi.org/10.1080/17441692.2025.2515481.
